# Canonical and Non-Canonical Autophagy in HIV-1 Replication Cycle

**DOI:** 10.3390/v9100270

**Published:** 2017-09-23

**Authors:** Olivier Leymarie, Leslie Lepont, Clarisse Berlioz-Torrent

**Affiliations:** 1 Inserm, U1016, Institut Cochin, 75014 Paris, France; olivier.leymarie@inserm.fr (O.L.); leslie.lepont@inserm.fr (L.L.); 2 CNRS, UMR8104, 75014 Paris, France; 3 University Paris Descartes, Sorbonne Paris Cité, 75006 Paris, France; 4 Institut Cochin, Department Infection, Immunity, Inflammation, 75014 Paris, France

**Keywords:** HIV-1, autophagy, LC3-associated phagocytosis, Env, Tat, Nef, Vpu, LC3

## Abstract

Autophagy is a lysosomal-dependent degradative process essential for maintaining cellular homeostasis, and is a key player in innate and adaptive immune responses to intracellular pathogens such as human immunodeficiency virus type 1 (HIV-1). In HIV-1 target cells, autophagy mechanisms can (i) selectively direct viral proteins and viruses for degradation; (ii) participate in the processing and presentation of viral-derived antigens through major histocompatibility complexes; and (iii) contribute to interferon production in response to HIV-1 infection. As a consequence, HIV-1 has evolved different strategies to finely regulate the autophagy pathway to favor its replication and dissemination. HIV-1 notably encodes accessory genes encoding Tat, Nef and Vpu proteins, which are able to perturb and hijack canonical and non-canonical autophagy mechanisms. This review outlines the current knowledge on the complex interplay between autophagy and HIV-1 replication cycle, providing an overview of the autophagy-mediated molecular processes deployed both by infected cells to combat the virus and by HIV-1 to evade antiviral response.

## 1. Introduction

Macroautophagy (herein called autophagy) is a lysosomal degradation pathway-dependent mechanism, initially described as being involved in the turnover of cellular components (proteins, lipids and organelles), either to maintain cell homeostasis or as a source of energy in response of metabolic stress. In the early 2000s, it became obvious that autophagy is also involved in immune response as a key element of innate defense against bacterial infection [1,2,3] or adaptive immunity by participating in major histocompatibility complex class II (MHC-II) processing of viral antigens [4]. Since then, numerous studies have provided evidence that autophagy plays a crucial role in host response, especially by selectively degrading intracellular pathogens or targeting cytosolic exogenous antigens to MHC-II containing compartment. The autophagic process also plays a part in the recognition of pathogen-associated molecular patterns (PAMPs) through Toll-like receptors (TLRs) by delivering cytosolic microbial genetic material to endosomal TLRs. Furthermore, autophagy acts as a downstream effector of pattern-recognition receptors (PRRs), signaling to eliminate intracellular pathogens or to regulate inflammatory response.

Human immunodeficiency virus (HIV), the etiologic agent responsible for AIDS, as an obligate intracellular pathogen, needs to circumvent cells’ intrinsic defenses and evade immune surveillance in order to efficiently propagate in infected patients. Interestingly, HIV infects CD4-expressing cells, such as CD4+ T lymphocytes (CD4+ T cells), macrophages and dendritic cells (DCs), leading to the failure of the immune system and resulting in a positive feedback loop for virus replication and spread. In addition, HIV can persist for a long time in these target cells. Thus, CD4+ T cells, macrophages and DCs form viral reservoirs, despite the fact that these cellular populations are among the most specialized host cells to detect and mount an immune response to eliminate pathogens. Since it is clear that the autophagy mechanism participates in numerous antiviral activities deployed by CD4-expressing immune cells, many studies have been carried out to evaluate the impact of autophagy on the HIV life cycle. This review discusses the intricate interplay between HIV and autophagy, highlighting how HIV exercises fine control over the autophagy pathway in its target cells.

## 2. An Overview of the Autophagy Mechanism

**Figure 1:**
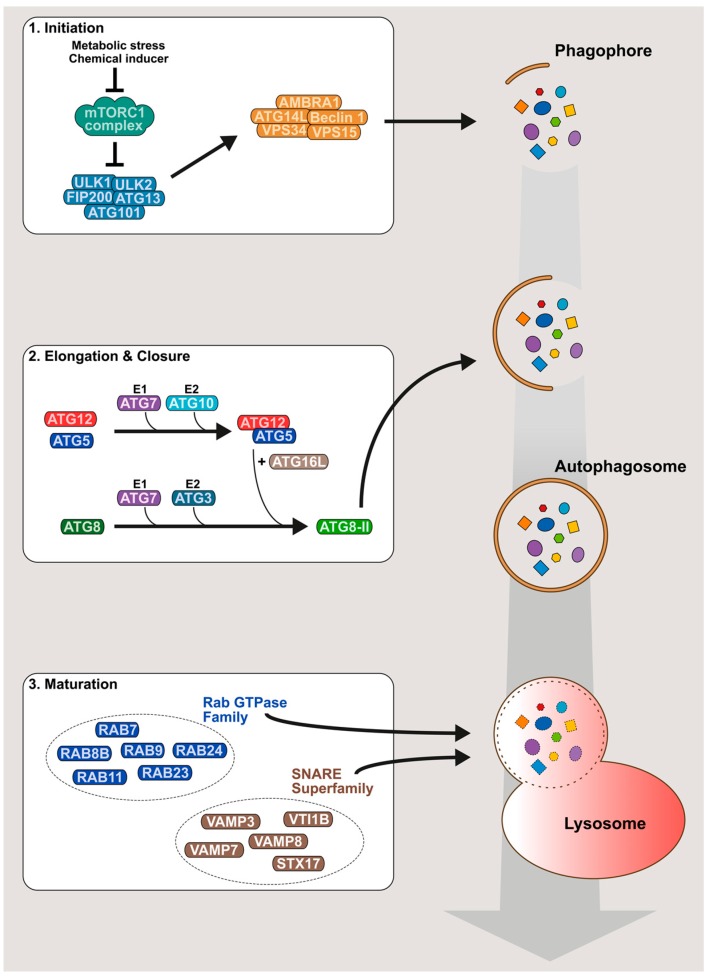
Schematic model of canonical autophagy. Canonical autophagy, which induces formation of double-layered membrane vesicles (autophagosomes) sequestrating cytoplasmic material, can be divided into three main stages: (1) initiation; (2) elongation and closure; and (3) maturation. Briefly, in the absence of pro-autophagy signals, mTORC1 complex maintains the preinitiation complex of autophagy (ULK1, ULK2, FIP200, ATG101 and ATG13) in an inactivated state by phosphorylating ULK1 and ATG13. Upon autophagy stimulation (metabolic stress or chemical inducers), mTORC1 complex is inhibited, allowing the preinitiation complex to activate the PI3K complex (VPS34, VPS15, Beclin 1, ATG14L and AMBRA1). This latter triggers the isolation of a cellular membrane (phagophore) at the endoplasmic reticulum (ER) surface by producing phosphatidylinositol-3-phosphate. Elongation and closure of phagophore are driven by lipidated forms of ATG8 family members. ATG8 orthologs exist in two forms, depending on the autophagy state: a non-lipidated cytosolic form (ATG8; inactive autophagy state) or a lipidated membrane-bound form (ATG8-II; active autophagy state). ATG8 lipidation reaction is controlled by two ubiquitine-like systems composed of the E1-like enzyme ATG7 and E2-like enzymes ATG10 or ATG3. The ATG7/ATG10 system generates the ATG5-ATG12 conjugate which, in interaction with ATG16L, is essential for specifying the site of ATG8-II production at the phagophore membrane. The maturation step, corresponding to the fusion between autophagosomes and lysosomes, is regulated by members of the Rab GTPase family and the SNARE superfamily. This process results in degradation of the autophagosomal content. ULK: UNC-51-like kinase; FIP200: focal adhesion kinase family-interacting protein of 200 kDa; mTORC1: mTOR complex 1; PI3K: phosphatidylinositol 3-kinase; VPS: vacuolar protein sorting proteins; AMBRA1: activating molecule in Beclin 1-related autophagy 1; ATG: autophagy-related proteins; SNARE: soluble *N*-ethylmaleimide attachment protein receptor.

In mammalian cells, canonical autophagy is regulated by approximately 30 autophagy-related proteins (ATG) and several non-ATG proteins, which coordinate the formation of intracellular vesicles, sequestering portions of cytoplasmic material in a three-step process: initiation, elongation and maturation (Figure 1). The initiation step starts with the isolation of a cellular membrane (called phagophore) at the endoplasmic reticulum (ER) surface by the formation of an omega-like shape protrusion [5,6,7]. This structure is induced by a preinitiation complex composed of UNC-51-like kinase (ULK) 1 and ULK2 proteins, a focal adhesion kinase family-interacting protein of 200 kDa (FIP200), and ATG101 and ATG13 proteins [8,9,10,11,12]. In the absence of stimuli, the preinitiation complex is inhibited by a key regulator of the autophagy machinery, the serine/threonine kinase mammalian target of rapamycin (mTOR) protein. mTOR, in complex with different cellular cofactors to form mTOR complex 1 (mTORC1), maintains the preinitiation complex in an inactivated state by phosphorylating ULK1 and ATG13 [10,11,12]. After stimulation by metabolic stress (e.g., starvation, oxidative stress or hypoxia) or chemical autophagy inducers such as rapamycin [13], mTORC1 is inhibited, allowing the release and the translocation of the preinitiation complex to the site of phagophore formation [14,15]. Subsequently, ULK1 activates the class III phosphatidylinositol 3-kinase (PI3K) complex formed by the PI3K vacuolar protein sorting 34 (VPS34), VPS15 (also called p150), Beclin 1/ATG6, ATG14L and the activating molecule in Beclin 1-related autophagy 1 (AMBRA1) protein. The activated PI3K complex is targeted to the ER-mitochondria contact sites, where it produces phosphatidylinositol-3-phosphate (PI(3)P), triggering phagophore formation [16,17,18,19]. The elongation step, corresponding to phagophore expansion, is regulated by the ATG5-ATG12 conjugate and lipidated forms of ATG8 family members [20,21,22,23]. Production of these components requires two ATG7-dependent ubiquitin-like conjugation systems. Indeed, the E1-like enzyme ATG7, in association with E2-like enzymes ATG10 or ATG3, promotes the formation of ATG5–ATG12 conjugate [24,25,26] or the lipidation of ATG8s with phosphatidylethanolamine (mostly designated ATG8-II or LC3-II) [27,28], respectively. ATG8 members, comprising LC3-like (LC3A, LC3B and LC3C) and GABARAP-like (GABARAP, GABARAPL1 and GABARAPL2) subfamilies, are key markers used to monitor the autophagy process [29]. At the steady state, ATG8 proteins exist as free cytosolic forms, usually referred to as ATG8-I or LC3-I. Once autophagy is induced, ATG8 proteins are translocated to the nascent phagophore, where the lipidation reaction tightly binds ATG8s to the membrane. The ATG5-ATG12 conjugate, in interaction with ATG16L, controls ATG8s lipidation by specifying the membrane localization where the reaction takes place [30]. ATG8 proteins drive membrane expansion [31] and phagophore closure [32]. The resulting double-layered membrane vesicle is called autophagosome. The maturation step corresponds to the fusion of autophagosomes with lysosomes to generate autolysosomes, where the autophagosomal content is degraded. This step is regulated by cellular machineries involved in vesicle transport and fusion. In particular, several studies have shown that members of the Rab GTPase family (RAB7, RAB8B, RAB9, RAB11, RAB23 or RAB24) [1,33,34,35,36] and the soluble *N*-ethylmaleimide attachment protein receptor (SNARE) superfamily (VAMP3, VAMP7,VAMP8, VTI1B or STX17) [36,37,38,39] are essential for the late stage of autophagy mechanism.

## 3. Cell Type Specific Modulation of Autophagy during HIV-1 Infection

**Figure 2:**
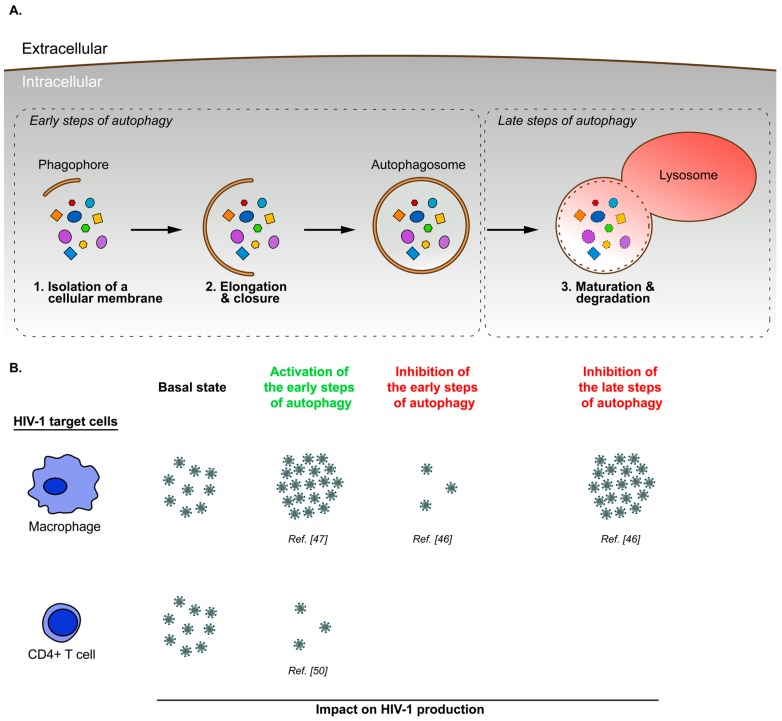
The interplay between autophagy and HIV-1 production in cell type specific manner. (**A**) Canonical autophagy mechanism can be decomposed into early steps, corresponding to the isolation, elongation and closure of a cellular membrane (phagophore) to form a double-layered membrane vesicle enclosing cytoplasmic materials (autophagosome); and late steps, corresponding to the autophagosome-lysosome fusion/maturation and the degradation of autophagosomal content; (**B**) In HIV-1 infected cells, activation or inhibition of early and late steps of autophagy process modulate virus production in a cell-type-specific manner. While induction of autophagy dramatically impairs HIV-1 production in CD4+ T cells, activation of early steps of autophagy is indispensable for the optimal production of viruses in macrophages. Conversely, blockage of late steps of autophagy favors an efficient HIV-1 production in macrophages.

Among HIV-infected patients, a few (around 5%) can control disease progression without antiretroviral therapy, remaining clinically asymptomatic and maintaining a normal CD4+ T cell count for at least 8 to 10 years [40,41]. These patients are divided into two groups, according to the viremia level: the viremic controllers (50–2000 HIV RNA copies/mL; VC) and the elite controllers (<50 HIV RNA copies/mL; EC) [42,43,44]. These two groups have been extensively studied to identify the cellular factors allowing viral control. A comparative analysis of peripheral blood mononuclear cells (PMBC) isolated from VC-, EC- and HIV-1-infected normal progressor donors underlined differences in autophagy pathway activity [45]. In particular, this study reports that VC and EC donors have a greater number of autophagic vesicles associated with an increased expression of autophagic markers with respect to normal progressors. In addition, rapamycin treatment of PBMC from VC donors induces an increased average number of autophagosomes per cell, where viral particles are trapped, an enhanced autophagic flux and a reduced virus production in comparison to untreated condition, while no modification is observed with normal progressors’ PBMC. HIV-1 could thus block the autophagy process even upon rapamycin treatment in normal progressors’ PBMC. Conversely, HIV-1 controllers seem to be able to bypass this block and sustain a fully functional autophagy mechanism engaged in viral particle elimination. However, this global analysis of autophagic response in infected PBMC does not take into account the cell-type-specific modulation of autophagy during HIV-1 infection. A more detailed examination of the heterogeneous population composing PBMC (lymphocytes, monocytes/macrophages and DC) reveals significant differences in the autophagic response to HIV-1 infection depending on the target cell (Figure 2).

In primary monocyte-derived macrophages (MDM), HIV infection induces the activation of the early steps of the autophagy mechanism and inhibition of the late stage of this process. This latter one is necessary for an optimal HIV-1 replication [46,47]. Interestingly, a transmission electron microscopy analysis carried out by Espert et al. highlights the fact that viral particles are only detected in weakly autophagic MDM [46]. These data suggest that HIV-1 maintains a delicate balance between activation and inhibition of the autophagy pathway to favor its replication in MDM.

In infected CD4+ T cells, the role of autophagy on HIV-1 production remains controversial. On the one hand, two studies report that productive HIV-1 infection of CD4+ T cells (primary cells or MOLT-X4 cell line) downregulates the autophagy pathway [46,48]. On the other hand, data presented by Laforge et al. indicate an increased expression of Beclin 1, ATG5 and LC3-II in HIV-1-infected primary CD4+ T cells compared to uninfected cells [49]. The use of different HIV-1 strains in these studies could explain those discrepancies. Interestingly, a more recent study showed that HIV-1 glycoprotein envelope (Env) triggered autophagy within the first 4 h following CD4+ T cell infection, and that autophagy is inhibited by HIV-1 48 h post-infection [50]. Accordingly, one may assume that, depending on the strain, HIV-1 cannot always invert the Env-mediated induction of autophagy in CD4+ T cells which could lead to an impaired HIV-1 replication in these cells. With regard to this, Laforge et al. [49] reveal that the induction of autophagy in response to the HIV-1 infection of CD4+ T cells is mediated by the damage-regulated autophagy modulator (DRAM), a lysosomal protein previously identified as a regulator of p53-induced autophagy [51]. In particular, they notice that depletion of DRAM increases the HIV-1 viral capsid (p24) present in the supernatant of infected CD4+ T cells. However, DRAM depletion also prevents HIV-1-mediated cell death, which does not allow a determination as to whether the p24 increase observed is due to a greater number of HIV-1 producer cells, or enhanced HIV-1 production. Nevertheless, the study by Sagnier et al. [50] demonstrated that treatment of primary CD4+ T cells or chronically-infected MOLT-X4 cells with Torin1, an mTOR inhibitor, impairs HIV-1 production. Consequently, this result suggests that autophagy activation has an antiviral effect in CD4+ T cells.

## 4. Autophagy-Mediated Viral Restriction in HIV-1 Infected Cells

To face viral infection, host cells possess a large array of “self-defense” mechanisms that constitute the intrinsic and innate immunity [52]. Intrinsic immunity is mediated by cellular pathways such as autophagy, RNA interference or may be conferred by a set of proteins, called restriction factors, which provide an immediate and direct antiviral activity. Conversely, innate immunity is an inducible mechanism, which is deployed less rapidly than intrinsic immunity, but results in a broader antiviral response. This mechanism is mediated by PRRs, such as TLRs, which detect virus-associated molecular patterns and trigger proinflammatory signaling cascades. In this section, we will highlight how autophagy pathway intersects with intracellular defense mechanisms to prevent HIV-1 infection. On the one hand, we will discuss the role of autophagy in the selective targeting to lysosomal degradation of viral components by proteins p62, the tripartite motif containing 5α (TRIM5α) and histone deacetylase 6 (HDAC6). On the other hand, we will examine the implication of autophagy in the induction of TLR-mediated proinflammatory response.

### 4.1. Degradation of the HIV-1 Tat Protein by the Selective Autophagy Mechanism

Although autophagy can be a non-specific degradation process, it can also specifically deliver cytosolic components to lysosomal degradation through the so-called selective autophagy mechanism. This mechanism requires autophagy receptors, which bridge selected cargo to nascent autophagosomes by interacting with the LC3-II proteins associated with the inner membrane of autophagosomes. Notably, interaction with LC3-II is mediated by LC3-interaction region (LIR) motifs present on autophagy receptors. One of the most extensively studied autophagy receptors is the p62 protein (also named sequestosome 1, SQSTM1), which is known to bind ubiquitinated substrates and to direct them for degradation [53]. Recently, p62 was reported to be involved in an autophagy-dependent degradation of the HIV-1 transactivator of transcription (Tat) protein [50]. Tat is an essential regulator of HIV-1 transcription by recruiting cellular transcription factors to the transactivation response element (TAR) presented within the 5′ end of viral transcripts [54]. In the study by Sagnier et al. [50], the authors revealed that p62 directly interacts with Tat in an ubiquitin-independent manner and mediates Tat degradation via the autophagic process. In particular, they showed that the transient Env-mediated induction of autophagy triggers this mechanism in CD4+ T cells, leading to impaired HIV-1 replication. In addition, their results indicate that secreted Tat proteins, known to affect gene expression and function in bystander cells, are similarly degraded in neighboring cells via selective autophagy.

### 4.2. Contribution of Autophagy in the TRIM5α Restriction Factor Activities against HIV-1

The p62 protein is also reported to participate in the anti-retroviral activity of the TRIM5α restriction factor. TRIM5α is a member of the TRIM family of proteins, which controls a large number of cellular processes (such as autophagy, innate immunity, apoptosis, or intracellular signaling). This restriction factor is also known to block HIV-1 infection by inducing a pro-inflammatory innate response and accelerating the uncoating of the viral core through binding to viral capsid [55,56,57]. More recent evidence highlights that TRIM5α can act as an autophagic receptor for HIV-1 p24, as well [58,59]. In particular, one study revealed that autophagy activation in HIV-1-infected rhesus monkey CD4+ T cells induced HIV-1 p24 degradation in a process depending on TRIM5α, p62, Beclin 1 and ATG7 [58]. The authors notably showed that mutating two LIR motifs in rhesus monkey TRIM5α (rhTRIM5α) prevents autophagy-mediated degradation of p24. Furthermore, on the basis that rhTRIM5α cannot bind simian immunodeficiency virus (SIV) capsid [57,60], Mandell et al. [58] demonstrated that autophagy induction hampers HIV-1 but not SIV infection, supporting the role of TRIM5α as an autophagy receptor for HIV-1 viral capsid. Some of the first investigations into human TRIM5α (huTRIM5α) found that this variant was much less efficient at blocking HIV-1 infection than rhTRIM5α [57,60,61,62]. However, recent work on Langerhans cells has suggested that huTRIM5α restriction efficacy may depend on autophagy process and the route of HIV-1 entry into host cells [59]. Langerhans cells form a subset of DC that is found in epidermis and mucosa. These cells are known to be the first line of defense against HIV-1 by capturing viruses via their cell surface C-type lectin receptor Langerin and degrading viral particles [63]. The study by Ribeiro et al. [59] revealed that depletion of Langerin, huTRIM5α, ATG5 or ATG16L in HIV-1 infected Langerhans cells leads to increased integration of viral DNA into the host genome. Conversely, HIV-1 integration is dramatically reduced after rapamycin treatment. Moreover, ectopic expression of Langerin in CD4+/CCR5+ U87 cells, a human glioblastoma cell line, also strongly inhibits HIV-1 infection in a huTRIM5α-, ATG16L-dependent manner. Interestingly, HIV-1 restriction activity of Langerin is not shared with the other C-type lectin receptor DC-SIGN (dendritic cell-specific ICAM-3-grabbing non-integrin). Indeed, depletion of huTRIM5α in DC-SIGN+ DC, a subset of DC known to be easily infected by HIV-1 [64], does not modify HIV integration and production. Finally, the authors showed that TRIM5α and ATG16L form a complex with Langerin through the binding of both TRIM5α and Langerin with leukocyte-specific protein 1 (LSP1). This led Ribeiro et al. to propose that, following binding to HIV-1, the Langerin-LSP1-TRIM5α-ATG16L complex is internalized into Birbeck granules, a specific compartment of Langerhans cells connected to the endosomal recycling pathway [65], which are subsequently delivered to nascent autophagosomes for degradation.

### 4.3. Modulation of Vif-Mediated Countermeasure against APOBEC3G Restriction by Autophagy

During the course of its evolution, HIV-1 has acquired accessory genes, encoding for proteins able to block cellular restriction factor activity. Among HIV-1 accessory proteins, the viral infectivity factor (Vif) binds and induces a proteasome-dependent degradation of the restriction factor apoplipoprotein B mRNA editing enzyme catalytic polypeptide-like 3G (APOBEC3G) [66]. This cellular factor was identified for both its ability to produce G to A hypermutations in the HIV-1 genome and to inhibit HIV-1 reverse transcription and proviral DNA integration [67,68,69,70,71]. Interestingly, it has been shown that HDAC6 is able to protect APOBEC3G against Vif-mediated degradation by interacting with APOBEC3G and/or inducing autophagy-dependent degradation of Vif, impairing HIV-1 infectivity [72]. This study has notably revealed that HDAC6 directly interacts with APOBEC3G and Vif, forming the ternary complex APOBEC3G-HDAC6-Vif or the two binary complexes HDAC6-APOBEC3G and HDAC6-Vif. By forming the HDAC6-APOBEC3G complex, HDAC6 prevents the association of APOBEC3G with Vif and, thus, inhibits the Vif-mediated ubiquitination and delivery of APOBEC3G to the proteasome. In addition, the HDAC6-Vif complex favors the autophagy-mediated degradation of Vif in a HDAC6 deacetylase activity and an ubiquitin-binding domain (BUZ domain)-dependent manner. HDAC6 deacetylase activity and BUZ domain have been linked to the clearance of polyubiquitin-enriched aggresome, a subcellular structure formed by the accumulation of polyubiquitin-positive misfolded proteins [73], by promoting fusion between lysosomes and aggresome-containing autophagosomes [74,75]. Thus, it can be hypothesized that HDAC6 promotes Vif degradation by favoring the association of Vif to the nascent aggresome-containing autophagosomes. In contrast with data by Valera et al. [72], a study indicates that inhibition of HDAC proteins, via HDAC chemical inhibitors (HDACi), promotes autophagy-mediated HIV-1 degradation in MDM by inhibiting the mTOR protein [76]. Although these studies might seem contradictory at first sight, HDAC family proteins differ widely in terms of sequence homology and cellular functions [77,78]. In addition, Campbell et al. [76] have not determined whether HDAC proteins directly act on cellular factors involved in mTOR regulation, or if HDAC proteins act on the transcription of autophagic factors related to their role as histone regulators. Thus, it is likely that HDAC family members differently regulate autophagy mechanism during HIV-1 infection.

### 4.4. Participation of Autophagy in the Induction of Innate Immunity to HIV-1 Infection

Recognition of HIV-1 by TLRs is also involved in the implementation of an efficient innate response to block HIV-1 spread. Particularly, it has been highlighted that the treatment of macrophages with two TLR8 agonists, ssRNA40 (a 20-mer phosphorothioate protected single-stranded RNA oligonucleotide) or the imidazoquinoline CL097, inhibits HIV-1 replication in an autophagy dependent manner in these cells [79]. This mechanism depends on the vitamin D signaling pathway, known to upregulate transcription of *Beclin 1* and *Atg5* in macrophages through the human cathelicidin antimicrobial peptide (CAMP)-dependent activation of the transcription factor CCAAT-enhancer-binding protein β (C/EBP-β) and the p38 mitogen-activated protein kinase (p38-MAPK) [80]. More precisely, in the presence of the active form of vitamin D, 1α,25-dihydroxycholecalciferol (1,25D3), and upon recognition of PAMPs, TLR8 induces *CAMP* gene upregulation, leading to an autophagy-mediated lysosomal-dependent inhibition of HIV-1 replication [79]. Autophagy is also implicated in the induction of interferon α (IFNα) by TLR7 in response to infectious or noninfectious HIV-1 or ssRNA40, in plasmacytoid DC (pDC) [81]. Indeed, ATG7 depletion by small interfering RNA (siRNA) or treatment with the autophagy inhibitor 3-methyladenine (3-MA) dramatically impedes IFNα production in pDC in the presence of HIV-1 particles. Interestingly, the authors revealed that 3-MA treatment during HIV-1 infection reduces IFN regulatory transcription factor 7 (IRF7) phosphorylation, which is necessary for the translocation of IRF7 to the nucleus and subsequent stimulation of IFNα expression.

## 5. Disruption of Autophagy-Associated Adaptive Immune Response by HIV-1

The establishment of a specific adaptive immune response to HIV-1 necessitates, in part, the processing and presentation of exogenous or endogenous viral-derived antigens through the MHC pathway in DC for an ensuing T cell activation and clonal expansion [82]. However, it has been shown that HIV-1 infection impedes DC maturation, leading to an impaired T-cell response [83,84,85]. In recent years, there has been growing evidence supporting the role of autophagy in the establishment of an effective adaptive immune response by participating in antigen processing and presentation [86]. Notably, autophagy can enhance antigen presentation by MHC molecules (class I or II) in antigen-presenting cells (APC) like DC or macrophages in response to microbial infection [87,88,89,90,91]. Interestingly, the study by Blanchet et al. [92] revealed that HIV-1 affects the presentation of exogenous antigens through MHC-II by downregulating autophagy flux initiation. Indeed, as early as 10 hours after exposure of monocyte-derived DC to viral particles, HIV-1 promotes mTOR activation, leading to a strong reduction of the DC-mediated CD4+ T cell response against HIV-1 antigens. This effect is not associated with any defects in DC maturation and cell surface MHC-II expression. Conversely, induction of autophagy with rapamycin in DC induced a robust CD4+ T cell response. These findings suggest that HIV-1-mediated inhibition of autophagy in DC might be a mechanism for evading adaptive immune response. In some circumstances, APC can also present endogenous viral antigens through MHC-II molecules. However, it has been highlighted that the role of autophagy in the MHC-II pathway is limited to exogenous antigen presentation. A recent study revealed that induction or inhibition of autophagy in HIV-1-infected DC do not alter endogenous viral antigen processing and presentation by MHC-II, and subsequent CD4+ T cell activation [83]. In addition, Blanchet et al. [92] reported that autophagy does not contribute to the cross-presentation process in DC, a mechanism allowing the processing and presentation of exogenous HIV-1 antigens by MHC-I molecules to CD8+ T cells.

The crucial role of autophagy in an effective MHC-II-mediated CD4+ T cell response was also confirmed in DC. Indeed, transduction of DC with a recombinant construct expressing a fusion protein formed by LC3B and the HIV-1 Gag precursor protein allows the specific targeting of this viral component to autophagosomes [83]. More interestingly, in an in vivo mouse model, expression of a SIV Gag protein fused to LC3B induces a stronger CD4+ T cell response-associated cytokine production (IFNγ, tumor necrosis factor α (TNFα) and interleukin 2 (IL-2)) and a higher number of SIV Gag-specific IFNγ-secreting CD4+ T cells in comparison with transduction of the SIV Gag protein alone [93]. Intradermal injection of mice with constructs expressing either the HIV-1 p24 alone or fused to the autophagy receptor p62 (p24/p62) led to very similar results. Thus, p24/p62-immunized mice displayed a higher number of p24-responding T cells, recognizing a more diverse p24 peptide repertoire, than p24-immunized mice [94]. Finally, the study by Yin et al. [93] revealed that the SIV-Gag-LC3B antigen induced a greater level of SIV Gag specific antibodies in mice compared to SIV Gag alone. This observation indicates that the contribution of autophagy in MHC-II-mediated CD4+ T cell activation also has important consequences for the establishment of an efficient humoral response against HIV-1.

## 6. Hijacking of Canonical Autophagy by HIV-1 Viral Proteins

Several HIV-1 proteins have been identified for their ability to alter the canonical autophagy pathway to promote viral production and propagation, evade the innate and adaptive immune system, or even favor pro-survival mechanisms to delay cell death. This section highlights the most documented HIV-1 proteins, demonstrating connections with canonical autophagy (Table 1).

### 6.1. Envelope Proteins

HIV-1 Env, which promotes viral entry by binding to cell surface receptor CD4 and co-receptor CXCR4 (CXC chemokine receptor 4) or CCR5 and fusing viral and plasma membranes, was the first HIV-1 viral protein identified for its ability to subvert the autophagy mechanism. Indeed, Env has been shown to induce autophagy-dependent apoptosis of uninfected bystander CD4+ T cells, likely significantly contributing to AIDS pathogenesis by depleting the CD4+ T cell population [95,96]. Env is a trimer of the heterodimer composed of non-covalently associated subunits gp120 and gp41, which are dedicated to viral receptor interaction and membrane fusion, respectively. In these studies [95,96], the authors revealed that the binding of gp120 with the CXCR4 co-receptor, but not CD4, is essential for induction of autophagy-mediated apoptosis. However, this process is not directly triggered by the gp120-CXCR4 interaction, but rather by the fusogenic activity of gp41. Indeed, a point mutation in the fusion domain of gp41 is sufficient to inhibit autophagy and the ensuing cell death of CD4+ T cells [96]. How Env-mediated autophagy induces apoptosis in uninfected CD4+ T cells has not been determined yet. Nevertheless, it appears that this mechanism requires a fully functional autophagy pathway since upon treatment with early or late autophagy inhibitors, such as 3-MA and bafilomycin A1 (a specific inhibitor of autophagosome-lysosome fusion) respectively, or following depletion of Beclin 1 and ATG7, uninfected CD4+ T cells do not undergo cell death following contact of Env [95]. Surprisingly, Env induces increased caspase-3 (a protease involved in cell death execution) activity in uninfected CD4+ T cells, treatment with the pan-caspase inhibitor z-VAD does not prevent Env-mediated apoptosis in these cells [95]. This result suggests that both caspase-dependent and caspase-independent apoptosis are involved in this mechanism.

In contrast to what is observed in CD4+ T cells, it has been shown that gp120 downregulates autophagy in DC [92]. This mechanism depends in part on the engagement of CD4 receptor with gp120, which results in Erk-mTOR signaling pathway activation and subsequent autophagy inhibition. Interestingly, this study revealed that HIV-1-mediated downregulation of autophagy in DC leads to a reduced DC-mediated CD4+ T cell response (as mentioned above), an impaired TLR signaling and an enhanced HIV-1 transmission to CD4+ T cells. Therefore, these observations suggest that gp120-mediated inhibition of autophagy in DC could promote viral spreading.

HIV-1 can infect macrophages and microglia in the brain, resulting in a progressive neurological injury of the central nervous system (CNS) which is due in part to the neurotoxic effect of gp120 [113]. Indeed, although neurons cannot be productively infected by HIV-1, virions or free-gp120 released from infected macrophages and microglia impact viability and biological functions of neurons [114]. Dysregulation of autophagy has been reported to contribute to various neurodegenerative diseases [115]. Interestingly, a higher expression of autophagy proteins (Beclin 1, ATG5, ATG7, LC3B-II) and a greater number of autophagosomes per neurons are found in post-mortem brain tissues from HIV-1-infected patients developing HIV-associated encephalitis (HIVE) than those with no HIVE [97]. Furthermore, several studies have demonstrated that treatment of neuroblastoma cells or astrocytes with free-gp120 induces autophagy [97,98,99,100]. Consequently, gp120-mediated autophagy induction in the CNS may be associated with neuronal damage. In addition, gp120 seems to have a mild to strong effect on neuroblastoma cell apoptosis, whereas no cell death induction is observed in gp120-treated astrocytes [98,99,100]. Two cellular factors have been identified to play a critical role in gp120-mediated autophagy in neuroblastoma cells, the apoptosis-stimulating protein of p53-2 (ASPP2) and proline oxidase (POX) proteins [98,99]. ASPP2 is induced in the presence of gp120, and positively or negatively regulates autophagy and apoptosis in a gp120 concentration-dependent manner [99]. Thus, while ASPP2 overexpression inhibits autophagy and apoptosis at low doses of gp120, it promotes activation of these cellular pathways at high doses of gp120. gp120 has also been shown to trigger autophagy by upregulating *POX* gene expression in a p53-dependent manner following its interaction with cell surface CXCR4 [98]. More specifically, engagement of CXCR4 with gp120 results in upregulation and nuclear translocation of p53. In turn, p53 recognizes and activates the *POX* gene promoter. Thereafter, POX, by catalyzing its substrate, generates reactive oxygen species (ROS), which induces autophagy in neuroblastoma cells. Why autophagy is induced in the presence of gp120 in neuronal cells remains to be determined. Nevertheless, it has been suggested that autophagy induction could function as a prosurvival response in these cells. This mechanism could be necessary to remove toxic stress and delay cell death, although contributing to neurological disorders [97,98].

### 6.2. Tat Protein

HIV-1 Tat has been shown to cause neuronal toxicity and dysfunction, and therefore seems to be a critical factor in HIV-1-associated neurocognitive disorder (HAND) [116,117,118,119]. In the brain, Tat is secreted by infected macrophages, astrocytes and microglia cells, but can also circulate in the bloodstream and cross the blood-brain barrier [120]. Tat can enter in neurons through endocytosis pathways which are mediated by numerous receptors such as CXCR4, CD26, heparin sulfate proteoglycans or low-density lipoprotein receptor-related proteins [121,122,123,124,125]. In the last few years, an increasing number of studies has found that Tat can modulate autophagy in brain tissues [101,102,103,104,105]. Interestingly, Tat regulates autophagy in a cell-type-dependent manner. It has been reported that Tat induces autophagy in human astrocyte cell lines and embryonic rat hippocampal neurons [103,105], and represses this mechanism in primary mouse neuron cells [104]. More surprisingly, Tat appears to have a dual role in autophagy in neuroblastoma cell lines depending on its concentration [101,102]. Indeed, autophagy is upregulated or downregulated at low or high doses of Tat, respectively. Moreover, while Tat reduces the number of autophagosomes and increases their size at low concentrations [102], it induces autophagosome accumulation and elicits the formation of misshapen autophagosomes at high concentration [101]. Interestingly, Fields and collaborators identified an interaction between Tat and lysosome-associated membrane protein 2 (LAMP2) [101], a lysosomal protein essential for chaperone-mediated autophagy and canonical autophagy [126,127]. Their work also revealed that Tat colocalizes with autophagosome and lysosomal markers and enhances autophagosome-lysosome fusion in neuroblastoma cells [101]. Therefore, the authors proposed that Tat, by interacting with LAMP2, may promote rapid and aberrant authophagosome-lysosome fusion, resulting in an accumulation of autophagolysosomes with altered morphology. Fields and collaborators have confirmed in vitro results by using an inducible Tat transgenic mouse model. Remarkably, they showed that Tat-mediated induction or repression of autophagy in neurons promote neurodegeneration [101,102]. In addition, they have demonstrated that Tat-mediated autophagy alteration can be reversed by treatment with autophagy inducers, reducing neuronal injury and improving locomotor activity in mice.

Tat also dysregulates autophagy mechanisms in macrophage/monocyte populations. In infected macrophages, Tat suppress IFNγ-mediated autophagy by inhibiting phosphorylation of signal transducer and activator of transcription-1 (STAT1) [106], a crucial signaling molecule downstream of IFNγ receptor. This block impairs autophagosome-mediated clearance of intracellular pathogens in macrophages, as illustrated by the loss of colocalization between IFNγ-induced autophagosomes and *Bacillus Calmette Guerin* in the presence of Tat. In a study by Van Grol et al. [107], Tat has been shown to inhibit autophagy in uninfected macrophages/monocytes by stimulating the Src-Akt signaling pathway, known to negatively regulate this mechanism [128,129,130]. More specifically, Tat activates Src-Akt signaling by interacting with the cell surface receptors CXCR4, vascular endothelial growth factor receptor (VEGFR) and β-integrins which are upstream of this pathway. This process also requires the STAT3 protein and concomitant activation of the IL-10 signaling pathway. Considering that Tat is secreted by infected macrophages, further investigation is needed in order to determine whether Tat modulates autophagy mechanisms in an autocrine manner as well.

### 6.3. Nef Protein

As mentioned above, autophagy is tightly regulated in macrophages by HIV-1. While early stages of this mechanism are essential for viral production, the degradation process is deleterious. In this respect, it has been highlighted that the HIV-1 negative regulatory factor (Nef) protein plays a pivotal role in the control of the late steps of autophagy. Nef is a multifunctional protein regulating several proteins with various functions in the cell [131]. Notably, Nef is well known for its ability to downregulate cell surface expression of CD4 and MHC complexes [132,133,134,135], and to antagonize the restriction factors serine incorporator 3 and 5 (SERINC 3/5) [136,137,138]. In 2009, Kyei et al. [47] identified an autophagy-inhibitory activity for Nef in macrophages. More specifically, they showed that Nef prevents autophagosome maturation and subsequent degradation of HIV-1. This effect is mediated by a direct or an indirect interaction of Nef with Beclin 1, and requires the Nef diacidic motif _174_DD_175_ [47] and the amino acid residues 267–284 in the evolutionarily conserved domain (ECD) of Beclin 1 [110]. Moreover, Nef is observed in intracellular structures that are positive for Beclin 1, ATG7 and ATG12. Interestingly, Nef-induced inhibition of autophagy appears to occur late in the course of macrophage infection; between days 3 and 5, post-infection [108]. Therefore, the authors proposed that Nef blocks autophagy once a productive infection of macrophages is established. They focused on the transcription factor EB (TFEB), a cellular protein that positively regulates the expression of several autophagy genes [139,140]. The authors particularly showed that, between 3 and 5 days post-infection, Nef suppresses the dephosphorylation of TFEB, preventing the ensuing nuclear translocation of this transcription factor, and hence inhibiting autophagy activity. In addition, Campbell et al. [108] revealed that, in the absence of Nef, HIV-1-induced TFEB translocation depends on Beclin 1, which is consistent with the Nef-Beclin 1 interaction identified previously. Note that, similarly to macrophages, Nef has also been shown to inhibit autophagosomes maturation in astrocytes [109], suggesting that Nef-mediated autophagy inhibition occurs in various cell types.

### 6.4. Vpr Protein

Very recently, a pro-autophagy activity was identified for the HIV-1 viral protein R (Vpr) [111]. More particularly, data revealed an increased level of LC3B and Beclin 1 in monocytic cell line-derived macrophages transiently expressing Vpr. Surprisingly, this increased level of autophagic markers is not accompanied by a greater number of autophagosomes in the cell, suggesting that only the early steps of autophagy are induced by Vpr. Interestingly, treatment with an early autophagy inhibitor promotes apoptosis in Vpr expressing macrophages. On the contrary, in the absence of Vpr, no increase in cell death is observed in early autophagy inhibitor-treated macrophages. Previous works in the literature noticed a dual role of Vpr in modulation of apoptosis in a cell type-dependent manner .Indeed, it has been shown that Vpr induces apoptosis in infected and/or uninfected lymphocytes, DCs, monocytes and neurons, but not in macrophages [141,142,143,144,145,146,147,148]. HIV-1-infected macrophages are known to be highly resistant to viral cytopathic effect and to have a prolonged lifespan [149,150,151,152,153], making these cells an HIV-1 reservoir. Thus, the role played by autophagy in the apoptotic properties of Vpr could be an important element in the disease progression. In this regard, it seems essential to further explore the interplay between Vpr, autophagy and apoptosis both in infected macrophages and in the other HIV-1 target cells.

## 7. Diversion of a Non-Canonical Autophagy Pathway, the LC3-Associated Phagocytosis, by HIV-1 Viral Protein Vpu

**Figure 3:**
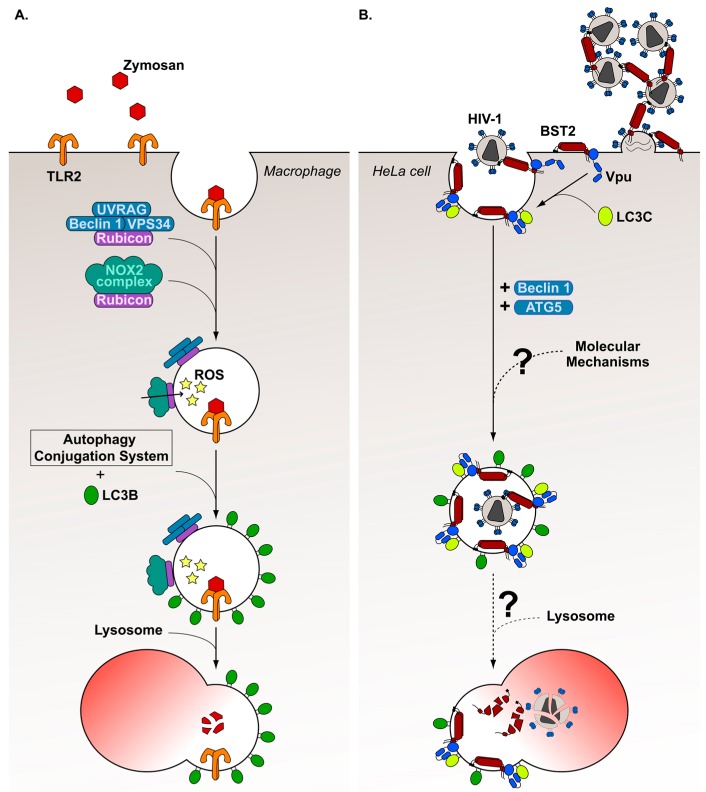
Comparative overview of LC3-Associated Phagocytosis (LAP)- and LC3C-dependent Vpu-mediated BST2 antagonism mechanisms. (**A**) Following its engagement with Toll-like receptor 2 (TLR2), zymosan is internalized by phagocytosis in macrophages. Subsequently, a PI3K complex composed of Rubicon, VPS34, Beclin 1 and UV radiation resistance-associated gene (UVRAG) proteins is recruited to the delimiting membrane of the phagosome resulting in a local production of phosphatidylinositol-3-phosphate (PI(3)P). This latter promotes recruitment of the NADPH oxidase-2 (NOX2) complex, which is responsible for reactive oxygen species (ROS) production into the phagosome. ROS, as along with the autophagy conjugation system, leads to decoration of the phagosome with LC3B, accelerating the phagosome-lysosome fusion process. (**B**) Following its interaction with the BST2 transmembrane domain at plasma membrane, Vpu recruits the LC3C protein to enhance internalization of BST2 into intracellular compartments, which are decorated with LC3 orthologs. This process requires Beclin 1 and ATG5 but not components of the preinitiation complex of autophagy (ULK1 and FIP200), which is reminiscent of the LAP mechanism, and results in the removal of BST2 from virus budding sites. However, the precise mechanisms by which this intracellular compartment is formed and the fate of this compartment within the cell are not yet determined.

Several non-canonical autophagy mechanisms have been identified in the past decade [154]. Among these, the LC3-Associated Phagocytosis (LAP) process was highlighted in 2007 by Sanjuan et al. [155]. The authors observed that TLR2 stimulation with zymosan, a yeast cell wall glucan, results in the engulfment of the TLR2-zymosan complex in a phagosome decorated with LC3-II molecules in macrophages (Figure 3A). This process depends notably on ATG5 and ATG7 proteins. In addition, the authors noticed a rapid Beclin 1 association with phagosomes, and an increased PI3K complex activity preceding LC3 recruitment. However, contrary to canonical autophagy, it was shown that LAP does not require all autophagy machinery components, since this process is independent of the autophagy preinitiation complex [156,157]. In a major advance in 2015, Martinez et al. [158] defined more precisely the molecular steps controlling the LAP mechanism, and identified the Rubicon protein as a LAP-specific marker. Thus, they revealed that, following zymosan internalization by phagocytosis, Rubicon is recruited at the delimiting membrane of the phagosome, resulting in local production of PI(3)P. Indeed, Rubicon interacts with a PI3K complex containing the UV radiation resistance-associated gene (UVRAG) protein, Beclin 1, VPS34, but not ATG14L or AMBRA1. Subsequently, PI(3)P mediates the recruitment of the NADPH oxidase-2 (NOX2) complex composed of NOX2 and the subunits p22PHOX, p40PHOX, p47PHOX and p67PHOX. This complex is stabilized at the phagosome membrane by a direct interaction between Rubicon and p22PHOX, inducing ROS production in the phagosome. ROS is demonstrated to be indispensable for LC3-II translocation at the phagosome membrane, although the precise mechanism has not been determined yet. The authors also demonstrated that components of the autophagy conjugation system (ATG5, ATG7, ATG12, ATG16L, ATG3 and ATG4B) are required for LC3-II recruitment. Finally, Martinez et al. [158] noticed that, by associating with phagosomes, LC3-II assists fusion between these structures and lysosomes, enhancing the maturation process.

Numerous studies have described the involvement of LAP in antibacterial [159,160,161,162,163,164,165,166], antifungus [167,168,169] and antiparasite [170,171,172] responses. Recently, our laboratory showed for the first time that a virus can hijack the LAP mechanism to promote viral spreading. Indeed, we demonstrated that the HIV-1 Vpu activity to counteract the cellular restriction factor bone marrow stromal cell antigen 2 (BST2) requires, in part, the LAP mechanism (Figure 3B) [112]. BST2 is a cell surface glycoprotein that is double-linked to cellular membranes through a *C*-terminus α-helical transmembrane domain and an extracellular N-terminus glycosylphosphatidylinositol anchor. This unusual protein structure allows BST2 to tether nascent viral particles to cellular membranes by inserting its extremities both into viral envelope and plasma membrane [173,174,175,176]. Therefore, BST2 prevents HIV-1 release, resulting in virus accumulation at the cell surface.

Prior to our recent study, some Vpu activities required to counterattack BST2 mediated restriction on HIV-1 budding had been highlighted. First, Vpu accelerates the targeting of BST2 to the lysosomal degradation pathway by promoting ubiquitination of the newly synthesized and/or recycling BST2 molecules trafficking through endosomes [177,178,179,180]. This mechanism leads to reduced BST2 recycling to the cell surface and BST2 cell surface downregulation. Independently, Vpu also favors HIV-1 release by interfering with BST2 trafficking via interaction of Vpu with clathrin adaptor protein complex-1 (AP-1) and AP-2 [181,182,183], or by directly removing BST2 from virus budding sites at the plasma membrane [184,185,186]. Our recent work reveals that Vpu can also counteract BST2 restriction by hijacking a non-canonical autophagy process, reminiscent of a LAP process [112]. Indeed, in HeLa cells infected with VSV-G (vesicular stomatitis virus glycoprotein)-pseudotyped HIV-1 viruses, we observed that depletion of Beclin 1, ATG5 and LC3C, but not of preinitiation complex components (ULK1 and FIP200), leads to reduced HIV-1 release in a BST2 dependent manner. We describe a direct interaction between Vpu and LC3C mediated by the Vpu _63_LVEM_66_ motif. Mutation in this motif also results in decreased HIV-1 release. Furthermore, LC3C depletion results in virus sequestration in intracellular single membrane-bound compartments very similar to LC3-associated phagosomes. Interestingly, we noticed that LC3C is involved in Vpu-mediated removal of BST2 from virus budding site without affecting the BST2 turnover at the cell surface. This observation indicates that the LAP-dependent Vpu-mediated removing of BST2 is independent of Vpu-mediated BST2 degradation.

## 8. Concluding Remarks

In the past decade, it has emerged that the autophagy pathway intersects with the HIV-1 life cycle. This review highlights the fact that autophagy acts as a double-edged sword in HIV-1 infection. Autophagy provides not only an integral part of intracellular defenses developed by infected cells to eliminate HIV-1 and mount a pro-inflammatory response, but also serves an essential role in the proper production and dissemination of this virus. HIV-1 finely regulates the autophagy pathway, thereby making the analysis of this intricate interplay challenging. Furthermore, it appears that the role of autophagy in HIV-1 infection is very closely related to the cell type, given that the early steps of autophagy are required for viral replication in macrophages, but are detrimental in CD4+ T cells. In line with this, the HIV-1 genome encodes both pro- and anti-autophagy proteins, some of which display a dual role in a cell type-dependent manner. The molecular mechanisms in which early and late steps of autophagy are engaged in these different cell types remains largely unclear, and thus requires further investigation in the future. Moreover, the time course of viral infection should also be taken into consideration in future studies, in order to better understand the interplay between HIV-1 and autophagy and to resolve some discrepancies observed in previous studies. Finally, the recent observation that HIV-1 hijacks a non-canonical autophagy mechanism to counteract an innate barrier to viral infection could notably provide some clues to understand the benefit of autophagy proteins for HIV-1 production. Further investigation will be needed to precisely decipher the engagement of the LAP process in viral production in HIV-1 target cells. Addressing all these questions should provide the basis for the development of new drug design strategies to combat the virus.

## Figures and Tables

**Table 1:** Non–autophagic and autophagic functions of HIV-1 Env, Tat, Nef and Vpu proteins.

HIV-1 Proteins	Non-Autophagic Functions	Autophagic Functions	References (Associated with Autophagy)
Env (gp120 and gp41)	Binds to HIV-1 receptor and coreceptors; mediates fusion between virus envelope and plasma membrane.	Induces apoptosis in CD4+ T cell; impairs dendritic cells-mediated CD4+ T cell response; increases HIV-1 spreading from dendritic cells to CD4+ T cells; reduces TLR signaling response; upregulates autophagy in neurons.	[92,95,96,97,98,99,100]
Tat	Regulates HIV-1 and cellular genes transcription; upregulates cell surface CXCR4 expression in uninfected CD4+ T cells; mediates T cell activation; impedes CD4+ and CD8+ maturation; alters mature CD8+ activity; modulates apoptosis.	Blocks IFNγ-mediated autophagy in macrophages/monocytes; inhibits autophagyin uninfected macrophages/monocytes by stimulating Src-Akt signaling pathway; alters autophagosome-lysosomes fusion in neurons.	[101,102,103,104,105,106,107]
Nef	Downregulates cell surface expression of CD4, MHC-I, MHC-II, CD28, T cell receptor/CD3 complex; prevents incorporation of restriction factors SERINC3/5 in budding viruses; modulates CD4+ T cell activation.	Prevents autophagy-mediated HIV-1 degradation by blocking autophagosome maturation in macrophages and astrocytes; blocks nuclear translocation of the pro-autophagy transcription factor TFEB in macrophages.	[47,108,109,110]
Vpr	Induces apoptosis in infected and/or uninfected T cells, DCs, monocytes and neurons; blocks the cell cycle at G2 phase; reactivates viral expression in latently infected cells; influences reverse transcription accuracy; regulates nuclear import of the HIV-1 pre-integration complex.	Prevents apoptosis in macrophages by diverting early steps of autophagy.	[111]
Vpu	Induces CD4 degradation; downregulates cell surface expression of MHC-I, CD1d, CCR7, CD40, CD81, SLAMF6; induces degradation of the restriction factor BST2; induces mistrafficking of BST2; drives displacing of BST2 from virus budding sites; depolarizes membrane potential by forming ion channel.	Drives displacing of BST2 from virus budding sites via a non-canonical autophagy mechanism (LC3-associated phagocytosis).	[112]

MHC: major histocompatibility complex; SERINC3/5: restriction factors serine incorporator 3 and 5; TFEB: transcription factor EB; CCR7: chemokine receptor 7; SLAMF6: SLAM family member 6; BST2: bone marrow stromal cell antigen 2; CXCR4: CXC chemokine receptor 4.
